# Predictors of quality of life in parents of children with rare diseases: a tertiary care center cross-sectional study in Saudi Arabia

**DOI:** 10.3389/fpubh.2026.1767604

**Published:** 2026-04-01

**Authors:** Abdullah Alkhani, Mariam M. Aleissa, Fahad Almsned, Albara Arefi, Sarah Alsaleh, Aya Mousli, Haifa Almuhanna, Nasser M. Alzain, Sami Alhaider, Raghad Alhuthil, Afaf Alsagheir

**Affiliations:** 1 Department of Pediatrics, King Faisal Specialist Hospital & Research Centre, Riyadh, Saudi Arabia; 2 College of Medicine, Alfaisal University, Riyadh, Saudi Arabia; 3 Public Health Lab, Public Health Authority, Riyadh, Saudi Arabia; 4 King Khaled Eye Specialist Hospital and Research Center, Riyadh, Saudi Arabia; 5 Research Program, Academic, Training, and Research Administration, Eastern Health Cluster, Dammam, Saudi Arabia; 6 Research Center, King Fahad Specialist Hospital-Dammam, Dammam, Saudi Arabia; 7 School of Systems Biology, George Mason University, Fairfax, VA, United States; 8 Department of Psychiatry, College of Medicine, Imam Abdulrahman Bin Faisal University, Dammam, Saudi Arabia; 9 General Administration of Medical Services, Ministry of Interior, Riyadh, Saudi Arabia

**Keywords:** caregivers, congenital adrenal hyperplasia, cystic fibrosis, Duchenne muscular dystrophy, Family Impact, PedsQL, quality of life, rare disease

## Abstract

**Purpose:**

This research aims to investigate the medical, socioeconomic, and cultural factors influencing the quality of life (QoL) in parents of children with cystic fibrosis (CF), congenital adrenal hyperplasia (CAH), and Duchenne muscular dystrophy (DMD).

**Methods:**

This cross-sectional single-centered study included parents of children diagnosed with CF, CAH, or DMD at King Faisal Specialist Hospital and Research Center, Riyadh, Saudi Arabia. The QoL was assessed using the PedsQL™ Family Impact Module.

**Results:**

A total of 107 parents participated (response rate: 77%), including CF (*n* = 40), CAH (*n* = 26), and DMD (*n* = 41). Fathers comprised 59.8% of respondents. 85% of fathers were employed compared to 15% in mothers. Consanguinity was reported in 77.6% of families. Diagnostic delays exceeding 1 year occurred in 46.7% of cases, particularly in DMD (82.9%). Only 10.3% of parents participated in support groups. Multi-variate analysis using a generalized linear model showed that frequent emergency department visits (>6 per year) and DMD diagnosis were predictors of lower QoL (*p* = 0.003) and (*p* = 0.004), respectively. Higher QoL was associated with maternal status (*p* = 0.045) and higher income (*p* = 0.014).

**Conclusion:**

Overall, the study found that parents of children with rare diseases experience a suboptimal QoL score, with parents of children with DMD faced the greatest challenges, suggesting targeted interventions like enhanced newborn screening and utilizing genetic testing for expedited diagnosis may be beneficial. The study also found that frequent emergency department visits negatively impacted QoL, suggesting that enhancing healthcare access through medical education, and the integration of telemedicine and home healthcare services might alleviate the burden faced by caregivers. Moreover, financial support to these families might play a role in enhancing their quality of life due to high prevalence of sole providers, and caregiver saturation. The low participation in support groups signals a gap in caregiver support that requires further exploration.

## Introduction

Rare diseases comprise a diverse group of conditions affecting fewer than one in 2,000 individuals. In some regions, definitions vary based on prevalence; for instance, in the United States, a disease is considered rare if it affects fewer than 200,000 people (1). Ultra-rare diseases on the other hand, are defined as diseases with prevalence less than one in 50,000 individuals (2). Approximately 7,000 rare diseases have been identified, with 80% attributed to genetic causes (3). These diseases encompass but are not restricted to, cystic fibrosis (CF), Duchenne muscular dystrophy (DMD), and congenital adrenal hyperplasia (CAH) (4–6).

Rare diseases have certain characteristics that make them unique and can lead to compounded effects. Firstly, rare diseases typically manifest in early childhood, leading to a prolonged and intensive period of medical visits and investigations before a definitive diagnosis is reached which is a process known as “diagnostic odyssey” (7). This process is also exacerbated by the gaps in knowledge among healthcare providers (8). Secondly, due to their low prevalence, fewer than 5% of rare diseases have approved treatments. This limited number is attributed to the small patient populations, which generally discourage pharmaceutical companies from investing in drug development, as the potential market may not be profitable enough to justify the costs (9, 10). Furthermore, despite Saudi Arabia’s advancement in leading complex health challenges in the region, rare diseases remain under-recognized within Saudi Arabia’s healthcare priorities (11).

The multi-dimensional aspects of rare diseases are important to understand when discussing the topic. The high risk of morbidity and mortality of rare diseases in children pose great deal of stress to families (12). In addition to that, social inequalities like poverty, discrimination, and work-related adversities can lead to non-medical challenges like education, employment, and social challenges later on (13). Moreover, the financial burden of rare diseases on the family or the government is substantially high and is heightened in the pediatric population. In fact, a study by Chung et al. showed that estimated expenditure for a pediatric rare disease patient in China was (US$840,908) per person per year compared to (US$324,126) in that of adults (14). Furthermore, the physiological burden to the child affected with rare disease and his/her family can impede daily function. For instance, a previous study showed that over 70% of individuals with rare diseases face difficulties with daily tasks and activities, and social interactions while 42% of caregivers dedicate 2 hours daily for disease-related activities like hygiene, treatment management, patient mobility, and housework assistance (15).

Saudi Arabia is a critical setting for examining rare diseases due to a high prevalence driven by consanguinity, which increases the frequency of pathogenic alleles (16–18). Moreover, studies from Saudi Arabia reported significant diagnostic delays and limited access to advanced genetic testing (19). While the geographic distribution of rare diseases varies across Saudi Arabia, tertiary care remains centrally organized through major referral hospitals serving extensive regions (20, 21). These all are important when understanding how rare diseases affect quality of life of the child and family.

Quality of life (QoL) is significantly impacted in families with a member affected by a rare disease. The World Health Organization (WHO) redefined health in 1948, stating that it is not merely the absence of disease but also the presence of physical, mental, and social well-being (22). Families affected by rare diseases typically experience emotional instability, heightened anxiety, depression, diminished social support, and overall reduced well-being. Both disease-specific factors, such as frequent hospitalizations, and psychosocial factors, encompassing motherhood and increasing parental age, are strongly associated with a decline in QoL (23, 24). Addressing such challenges requires a multifaceted approach involving individual, community, and governmental interventions to support affected families properly (25, 26). To capture the multifaceted burden on caregivers, we utilized the PedsQL™ Family Impact Module, which is a validated, reliable, multidimensional instrument capable of distinguishing between multiple quality of life dimensions including physical, emotional, and social well-being in the context of chronic pediatric conditions (27).

Understanding the intersection between rare diseases and quality of life (QoL) may help healthcare systems better support affected children and their families. Therefore, this study aims to investigate the medical, socioeconomic, and cultural factors influencing QoL in parents of children with CF, CAH, or DMD. These three conditions were selected to represent rare pediatric diseases with variation in prevalence, clinical burden, diagnostic and treatment approaches, newborn screening availability, and disease trajectory.

## Methods

This cross-sectional observational study examined parents of children diagnosed with rare diseases from July 2019 to July 2024 at King Faisal Specialist Hospital and Research Center in Riyadh, Saudi Arabia. This period encompasses the COVID-19 pandemic which might have affected access to care and overall quality of life of parents. Data were collected via an electronic questionnaire. While this method allowed for efficient data capture, we acknowledge the potential for selection bias, as participation was limited to parents with digital literacy and access to electronic devices. The questionnaire included relevant variables and the PedsQL™ Family Impact Module—Parent Report in its Arabic version (27). Permission for its use was obtained from the original author.

To encourage participation, a reminder message was sent to non-responders via call or message 1 week after the initial invitation, with a single follow-up. Participation was voluntary, and respondents could withdraw at any time by exiting the survey without submitting their responses. All responses were anonymized to ensure confidentiality.

### Eligibility criteria

All parents of pediatric patients under 18 years old with a confirmed diagnosis of CF, CAH, or DMD within the past 5 years at King Faisal Specialist Hospital and Research Center were included. Parents of children who had passed away, had an unconfirmed diagnosis, or received treatment outside Saudi Arabia were excluded. Even though parents of undiagnosed children face severe “diagnostic odyssey” and decline in quality of life, we opted to have the independent variable as a confirmed rare disease to compare clinical trajectories.

### Instrument

The PedsQL™ Family Impact Module is a parent self-report instrument that measures the impact of a child’s chronic illness on parents and families. This adaptation has established reliability and validity for use in pediatric populations. In the current study, the Arabic version demonstrated high internal consistency, with a Cronbach’s alpha of 0.847, further confirming its reliability within the Arabian context (28). It assesses health related QoL and family functioning across eight scales: Physical Functioning (six items), Emotional Functioning (five items), Social Functioning (four items), Cognitive Functioning (five items), Communication (three items), Anxiety (five items), Daily Activities (three items), and Family Relationships (five items). Parents rate each item on a 5-point Likert scale (0 = never a problem, 4 = almost always a problem). The scores from the 36 items are averaged to generate a total PedsQL™ Family Impact Module score, which is then transformed to a 0-to-100 scale (0 = almost always a problem, 100 = never a problem).

### Independent variables

External questions were added as independent variables to capture clinical predictors (diagnosis, healthcare utilization, timeline, family medical history), socioeconomic predictors (employment, salary, perceived obstacle to care), and demographic and cultural predictors (caregiver, consanguinity, geographic region, family structure).

### Missing responses

Responses with missing data (*n* = 2, 1.8%) were excluded from the analysis.

### Data analysis

Analyses were done using R. The reliability of the PedsQL™ questionnaire was measured using Cronbach’s alpha, revealing a strong internal consistency score of 0.847.

To investigate predictors of QoL scores, the one-way analysis of variance (ANOVA) was conducted with Tukey’s HSD *post hoc* test to identify significant group differences, followed by multivariable analysis using the generalized linear model (GLM) with stepwise regression. We employed a multivariable stepwise regression (generalized linear model) as an exploratory approach. This allowed us to identify the most significant predictors of QoL from a broad set of clinical, socioeconomic, and cultural variables in an understudied population where established theoretical models are limited.

## Results

### Study participants

A total of 141 eligible parents were contacted, and 109 responses were collected yielding a response rate of 77%; however, two responses were omitted due to incomplete responses. The distribution of participants was as follows: CF (*n* = 40), CAH (*n* = 26), or DMD (*n* = 41). Fathers accounted for 59.8% of the responders, while mothers made up 40.2%. Among children with rare diseases, 65.4% were male, and 34.6% were female, with 95.1% of DMD patients being males. The Eastern region showed a notably high concentration of CF cases, accounting for 37.5% of the total CF cohort compared to other regions (Table 1).

**Table 1:** Baseline demographic, socioeconomic, cultural, and disease-related characteristics of the study population by diagnosis.

Variable	Response	CAH (*n* = 26)	CF (*n* = 40)	DMD (*n* = 41)	Total (*n* = 107)
Responder to the PedsQL™	Father	9 (34.6)	27 (67.5)	28 (68.3)	64 (59.8)
	Mother	17 (65.4)	13 (32.5)	13 (31.7)	43 (40.2)
Gender of affected child	Male	12 (46.2)	19 (47.5)	39 (95.1)	70 (65.4)
	Female	14 (53.8)	21 (52.5)	2 (4.9)	37 (34.6)
Region	Central	7 (26.9)	6 (15.0)	13 (31.7)	26 (24.3)
	Eastern	0 (0.0)	15 (37.5)	5 (12.2)	20 (18.7)
	Northern	14 (53.8)	8 (20.0)	11 (26.8)	33 (30.8)
	Southern	3 (11.5)	6 (15.0)	6 (14.6)	15 (14.0)
	Western	2 (7.7)	5 (12.5)	6 (14.6)	13 (12.1)
Employed father	Yes	21 (80.8)	35 (87.5)	34 (82.9)	90 (84.1)
	No	5 (19.2)	5 (12.5)	7 (17.1)	17 (15.9)
Employed mother	Yes	5 (19.2)	5 (12.5)	6 (14.6)	16 (15.0)
	No	21 (80.8)	35 (87.5)	35 (85.4)	91 (85.0)
Monthly salary (SAR)	Less than 4,000	3 (11.5)	14 (35.0)	13 (31.7)	30 (28.0)
	4,000–9,999	17 (65.4)	18 (45.0)	11 (26.8)	24 (22.4)
	10,000–20,000	3 (11.5)	7 (17.5)	14 (34.1)	46 (43.0)
	More than 20,000	3 (11.5)	1 (2.5)	3 (7.3)	7 (6.5)
Marital status	Married	25 (96.2)	36 (90.0)	39 (95.1)	100 (93.5)
	Divorced	1 (3.8)	3 (7.5)	0 (0.0)	4 (3.7)
	Father widowed	0 (0.0)	1 (2.5)	1 (2.4)	2 (1.9)
	Mother widowed	0 (0.0)	0 (0.0)	1 (2.4)	1 (0.9)
Sequence of the child	Only child	3 (11.5)	0 (0.0)	2 (4.9)	5 (4.7)
	Youngest child	11 (42.3)	22 (55.0)	14 (34.1)	47 (43.9)
	Middle child	10 (38.5)	11 (27.5)	14 (34.1)	35 (32.7)
	Oldest child	2 (7.7)	7 (17.5)	11 (26.8)	20 (18.7)
Number of siblings	One sibling	3 (11.5)	0 (0.0)	2 (4.9)	5 (4.7)
	Two siblings	3 (11.5)	4 (10.0)	2 (4.9)	9 (8.4)
	Three siblings	9 (34.6)	10 (25.0)	8 (19.5)	27 (25.2)
	Four siblings	1 (3.8)	8 (20.0)	5 (12.2)	14 (13.1)
	Five siblings	6 (23.1)	6 (15.0)	3 (7.3)	15 (14.0)
	Six siblings	1 (3.8)	9 (22.5)	9 (22.0)	19 (17.8)
	More than six siblings	3 (11.5)	3 (7.5)	12 (29.3)	18 (16.8)
Consanguinity	Yes	24 (92.3)	37 (92.5)	22 (53.7)	83 (77.6)
	No	2 (7.7)	3 (7.5)	19 (46.3)	24 (22.4)
ED visits per year	Less than three times	12 (46.2)	20 (50.0)	28 (68.3)	60 (56.1)
	Three to six times	7 (26.9)	13 (32.5)	8 (19.5)	28 (26.2)
	More than six times	7 (26.9)	7 (17.5)	5 (12.2)	19 (17.8)
Inpatient admission per year	Less than two times	21 (80.8)	29 (72.5)	38 (92.7)	88 (82.2)
	Two to four times	5 (19.2)	9 (22.5)	1 (2.4)	15 (14.0)
	More than four times	0 (0.0)	2 (5.0)	2 (4.9)	4 (3.7)
PICU admission per year	Less than two times	24 (92.3)	36 (90.0)	40 (97.6)	100 (93.5)
	Two to four times	1 (3.8)	3 (7.5)	1 (2.4)	5 (4.7)
	More than four times	1 (3.8)	1 (2.5)	0 (0.0)	2 (1.9)
Onset of symptoms to diagnosis	Newborn screening	2 (7.7)	2 (5.0)	0 (0.0)	4 (3.7)
	Antenatal diagnosis	16 (61.5)	4 (10.0)	0 (0.0)	20 (18.7)
	Less than 3 months	5 (19.2)	10 (25.0)	4 (9.8)	19 (17.8)
	Four to 6 months	1 (3.8)	7 (17.5)	0 (0.0)	8 (7.5)
	Six to twelve months	1 (3.8)	2 (5.0)	3 (7.3)	6 (5.6)
	More than a year	1 (3.8)	15 (37.5)	34 (82.9)	50 (46.7)
Family member with the same diagnosis	Yes	15 (57.7)	24 (60.0)	19 (46.3)	54 (50.5)
	No	11 (42.3)	16 (40.0)	22 (53.7)	53 (49.5)
Engagement in the support groups	Yes	0 (0.0)	6 (15.0)	5 (12.2)	11 (10.3)
	No	26 (100)	34 (85.0)	36 (87.8)	96 (89.7)
Biggest obstacle to care	Financial	10 (38.5)	11 (27.5)	8 (19.5)	29 (27.1)
	Geographical	9 (34.6)	19 (47.5)	4 (9.8)	32 (29.9)
	Medical	4 (15.4)	10 (25.0)	28 (68.3)	42 (39.3)
	Physical	1 (3.8)	0 (0.0)	1 (2.4)	2 (1.9)
	Social	2 (7.7)	0 (0.0)	0 (0.0)	2 (1.9)

CAH, congenital adrenal hyperplasia; CF, cystic fibrosis; DMD, Duchenne muscular dystrophy; SAR, Saudi Arabian Riyals; ED, emergency department; PICU, pediatric intensive care unit. Data are presented as frequencies (%).

Socially, large family structures were common with 48.6% of families having more than 4 siblings. Notably, 50.5% of families had at least one other member with the same diagnosis suggesting a state of caregiver saturation with the household micro-system. Furthermore, while 84.1% of fathers were employed, only 15% of mothers held jobs suggesting high dependency on single source of outcome. 43% of families reported a monthly income between 10,000–20,000 SAR, while 28% earned less than 4,000 SAR compounding the effect of single source of income and highlighting the financial burden in families with rare diseases. More than 6 ED visits per year was found in 17.8% of participants. Notably, consanguinity was reported in 77.6% of the families, with the highest prevalence found in the CF group at 92.5%. This might be linked to higher prevalence of autosomal recessive mutations leading to different phenotypes. The biggest obstacle to care was medical for DMD (68.3%), geographical for CF (47.5%), and financial for CAH (38.5%) (Table 1).

Nearly half (46.7%) of the parents experienced a diagnostic delay of more than 1 year from symptom onset, primarily 82.9% of DMD cases. Additionally, only 10.3% of the parents participated in support groups related to their child’s condition suggesting different support systems in these families. Among parents of children with DMD, 68.3% identified the lack of adequate medical treatment as the most significant barrier to care (Table 1).

### Predictors of QoL

**Figure 1:**
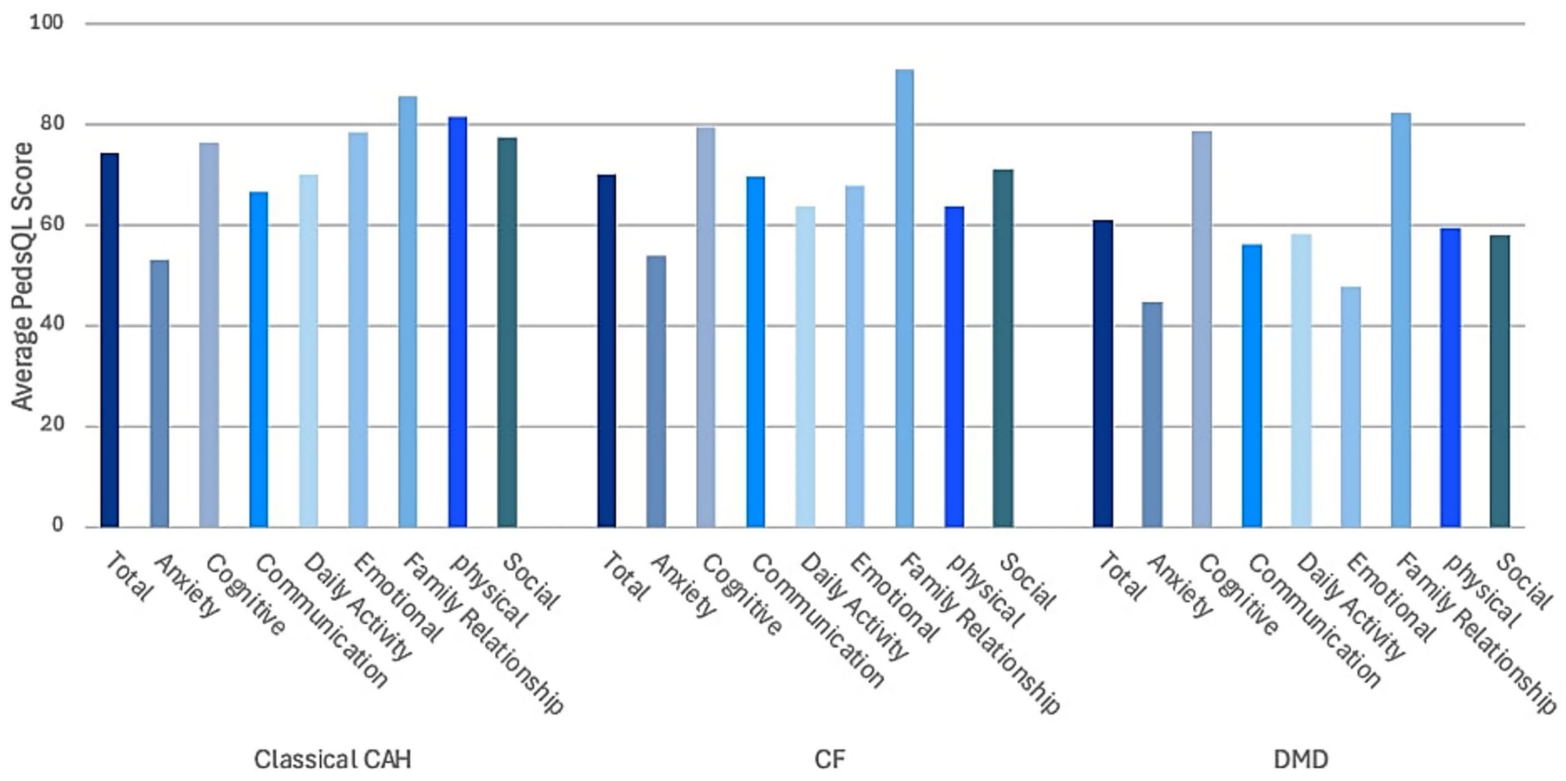
Domain-specific and total PedsQL™ scores reported by parents of children with congenital adrenal hyperplasia (CAH) (*n* = 26), cystic fibrosis (CF) (*n* = 40), and Duchenne muscular dystrophy (DMD) (*n* = 41).

The domain-specific PedsQL™ scores by disease were analyzed. The family relationship domain scored the highest among CAH and CF groups (85.6 and 90.8), respectively. While in the DMD group, the cognitive domain (78.7) scored the highest. Interestingly, anxiety domain scored the lowest in all groups (CAH: 53.1, CF: 54, DMD: 78.7) (Figure 1).

For CAH, the highest domain score is family relationship (85.6), indicating strong familial support, while anxiety scoring the lowest (53.1), suggesting higher stress levels among parents. Similarly, in CF, family relationship (90.8) is the highest, reflecting strong family support, whereas Anxiety (54) is the lowest, indicating elevated parental stress. In DMD, cognitive (78.7) scores the highest, suggesting better cognitive aspects of quality of life, while anxiety (44.9) is the lowest, highlighting significant parental stress and concern (Figure 1).

Univariate analysis analyzing factors significantly influencing lower parental QoL included DMD diagnosis (*p* = 0.025), male gender of the affected child (*p* = 0.031) likely confounded by DMD diagnosis as 95.1% of DMD patients were male, frequent emergency department (ED) visits (more than six visits per year) (*p* = 0.026), financial obstacles (*p* = 0.035), and paternal status (*p* = 0.019). Consanguinity was positively associated with higher QoL (*p* = 0.029). However, our univariate analysis, controlling for the False Discovery Rate (FDR) using the Benjamini-Hochberg procedure, revealed high false positive rate of 19.3% and should be flagged as exploratory findings (Table 2).

**Table 2:** Univariable analysis of factors influencing the quality of life of parents of children with rare diseases.

Variable	Response	Total mean **±** SD	*p*-value	*Post Hoc* pairwise comparison (mean difference, adjusted *P*-value)	Q (BH-FDR)	Effect size (interpretation)
Diagnosis	CAH	74.4 ± 20.5	0.025*	DMD vs. CAH: −13.3, *P* = 0.029*	0.193	0.069 (medium)
	CF	70.2 ± 20.5		CF vs. CAH: −4.2, *p* = 0.701		
	DMD	61.1 ± 20.3		DMD vs. CF: −9.1, *p* = 0.114		
Responder to the PedsQL™	Father	63.9 ± 21.7	0.019*	Mother vs. Father: 9.6, *P* = 0.019*	0.193	0.052 (medium)
	Mother	73.5 ± 18.7				
Gender of affected child	Male	64.6 ± 21.9	0.031*	Male vs. Female: −9.1, *P* = 0.031*	0.193	0.044 (small to medium)
	Female	73.7 ± 18.0				
Geography	Central	65.5 ± 23.9	0.454	–	0.701	0.035
	Eastern	63.2 ± 19.5		–		
	Northern	73.1 ± 18.4		–		
	Southern	68.6 ± 21.3		–		
	Western	64.6 ± 22.9		–		
Employed father	Yes	67.5 ± 21.5	0.777	–	0.939	0.001
	No	69.1 ± 18.4		–		
Employed mother	Yes	76.7 ± 22.0	0.064	–	0.231	0.032
	No	66.2 ± 20.5		–		
Monthly salary (SAR)	<4,000	60.3 ± 22.2	0.106	–	0.317	0.057
	4,000–9,999	73.8 ± 19.6		–		
	10,000–20,000	69.4 ± 19.6		–		
	>20,000	68.5 ± 24.1		–		
Marital status	Married	67.0 ± 21.2	0.523	–	0.757	0.021
	Divorced	81.9 ± 19.0		–		
	Father widowed	70.1 ± 15.7		–		
	Mother widowed	79.2 ± N/A		–		
Birth order of child	Only child	54.2 ± 20.5	0.459	–	0.701	0.025
	Youngest child	67.5 ± 22.5		–		
	Middle child	68.1 ± 19.3		–		
	Oldest child	71.1 ± 20.2		–		
Consanguinity	Yes	70.1 ± 20.4	0.029*	Yes vs. No: 10.6, *P* = 0.029*	0.193	0.044 (small to medium)
	No	59.5 ± 21.4				
ED visits	<3 times	72.0 ± 17.7	0.026*	>6 vs. <3: −14.4, *p* = 0.023*	0.193	0.068 (medium)
	3–6 times	65.6 ± 23.5		3–6 vs. <3: −6.4, *p* = 0.366		
	>6 times	57.6 ± 23.7		>6 vs. 3–6: −8.0, *p* = 0.387		
Inpatient admissions	<2 times	70.0 ± 19.5	0.032*	<2 vs. 2–4 Times: 9.9, *p* = 0.204	0.193	0.064 (medium)
	2–4 times	60.1 ± 22.7		>4 vs. 2–4 Times: −12.9, *p* = 0.504		
	>4 times	47.2 ± 34.2		>4 vs. <2 Times: −22.8, *p* = 0.081		
PICU admissions	<2 times	68.3 ± 20.0	0.138	–	0.366	0.037
	2–4 times	51.5 ± 35.0		–		
	>4 times	81.9 ± 17.7		–		
Onset to diagnosis	Newborn screening	69.1 ± 23.4	0.760	–	0.925	0.025
	Antenatal diagnosis	76.2 ± 19.9		–		
	<3 months	71.6 ± 22.4		–		
	4–6 months	70.8 ± 17.5		–		
	7–12 months	62.0 ± 27.5		–		
	>1 year	65.3 ± 19.7		–		
Family member with same diagnosis	Yes	67.3 ± 20.7	0.810	–	0.951	0.001
	No	68.2 ± 21.4		–		
Support group	Yes	67.4 ± 17.0	0.959	–	0.976	0
	No	67.8 ± 21.5		–		
Obstacle to care	Financial	59.7 ± 22.9	0.035*	Geographic vs. Financial: 16.8, *P* = 0.014*^a^	0.193	0.096 (medium to large)
	Geographical	76.5 ± 19.7				
	Medical	66.6 ± 19.1				
	Physical	64.2 ± 14.2				
	Social	71.9 ± 19.2				

CAH, congenital adrenal hyperplasia; CF, cystic fibrosis; DMD, Duchenne muscular dystrophy; SAR, Saudi Arabian Riyals; ED, emergency department; PICU, pediatric intensive care unit. *Statistically significant results (*p* < 0.05).

a Only the Geographic vs. Financial comparison was significant among obstacle-to-care categories; other comparisons were not statistically significant.

Multivariable stepwise regression analysis revealed that maternal status and having a higher income (10,000–20,000 SAR) were independently associated with higher QoL scores (*p* = 0.045 and *p* = 0.014, respectively). Conversely, increased ED visits had a negative impact on QoL, with parents of children requiring more than six visits per year reporting significantly lower QoL (*p* = 0.003). Additionally, parents of children with DMD experienced significantly lower QoL compared to other groups (*p* = 0.004) (Table 3).

**Table 3:** Multivariable stepwise regression analysis of factors affecting parental quality of life.

Variable	Estimate	Standard error	*T*-value	*P*-value
Responder mother	8.025	3.944	2.035	0.045^*^
Three to six ED visits/year	−7.712	4.416	−1.746	0.084
More than six ED visits/year	−16.302	5.243	−3.109	0.003^*^
Diagnosis, cystic fibrosis	−2.663	5.14	−0.518	0.606
Diagnosis, DMD	−15.713	5.381	−2.92	0.004^*^
Monthly salary 10,000–20,000 SAR	13.195	5.268	2.505	0.014^*^
Monthly salary 4,000–9,999 SAR	6.801	4.673	1.455	0.149
Monthly salary more than 20,000 SAR	4.295	8.164	0.526	0.600
Number of siblings	1.615	1.081	1.495	0.138

ED, emergency department; DMD, Duchenne muscular dystrophy; SAR, Saudi Arabian Riyals. * Statistically significant results (*p* < 0.05).

## Discussion

Our research explored the predictors of QoL in parents of children with rare diseases. Our findings highlight several points worth discussing.

Overall, the study found that parents of children with rare diseases experience suboptimal QoL scores with parents of children having DMD having the lowest QoL scores. Reduced QoL among caregivers of DMD patients has been previously reported (29, 30). Our multi-variate analysis revealed DMD diagnosis as a predictor of low quality of life (Table 3). This can be due to limited treatment modalities, late diagnosis, and disease burden. DMD classical management includes multi-disciplinary care and corticosteroid therapy having a life expectancy of 28 years. Nevertheless, new emerging gene therapies like Elevidys offer more promising life expectancy in subgroup of DMD patients, but costs, accessibility, and time of diagnosis which reflects muscle preservation remain a barrier (31–33). 82.9% of our DMD patients had over 1-year period from onset of symptoms to diagnosis. Delayed diagnosis was most common in children with DMD, correlating with the lowest QoL scores. For a total cohort where 69.1% rely on a single income, this prolonged diagnostic odyssey is not just a medical delay, but a sustained period of economic and psychological depletion. This is where expanding national newborn screening and utilizing genetic testing to expedite diagnosis can alleviate parental stress and shorten the “diagnostic odyssey (25).” The feasibility of newborn screening for DMD has been tested in the United States, showing promising results (34). Thus, implementing a similar screening program in Saudi Arabia might facilitate early diagnosis and improve clinical outcomes and parental well-being. Single-gene testing remains the primary diagnostic tool for most monogenic rare diseases (35).

Children with DMD often require rehabilitation, and a family-centered care approach has been shown to optimize disease management and improve family QoL (36). As with most rare diseases, long-term follow-up with multiple healthcare services is necessary, highlighting the role of multidisciplinary teams. Establishing dedicated centers for holistic management enhances both the child’s health and parental QoL (37).

It is important to note that in the univariate analysis, male gender was a significant predictor of lower QoL (*p* = 0.031). However, this is likely confounded by the diagnosis, as 95.1% of DMD patients in our cohort were male due X-linked recessive mode of inheritance in majority of cases. This confounding effect is further supported by the fact that gender did not remain significant in the multivariable analysis.

Consanguinity was notably higher in our cohort (77.6%) than in the general population (57.7–56%) (38, 39). This higher rate may be due to the prevalence of single-gene defects in consanguineous marriages (40). In CF, consanguinity can lead to genetic variants that are not amenable to CFTR modulator treatments (41, 42). CF patients had the highest consanguinity rate (92.5%) and notably higher PedsQL™ Family Relationship domain scores (average: 90%). Notably, our post-hoc pairwise analysis revealed significant association between consanguinity and better quality of life (*p* = 0.029) with small to medium effect size (0.044) yet high false positive rate (19.3%). Our finding of a positive association between consanguinity and QoL is exploratory and requires cautious interpretation. While consanguinity is clinically associated with increased genetic risk, its positive association with QoL in this cohort is likely explained by cultural factors where consanguineous marriages have been reported to have stronger family support and increased social cohesion (43). However, this remains an exploratory finding requiring further validation due to the high false discovery rate. Notably, nearly half of our participants had a family history of a similar disease, underscoring the importance of genetic counseling (44).

According to a 2018 report by the General Authority for Statistics, the average salary of Saudi citizens across all sectors is approximately 10,000 SAR (45). In our multi-variate analysis, financial stability significantly influenced QoL, with an income range of 10,000–20,000 SAR predicting better outcomes (*p* = 0.014). Despite only 24.3% of participants were living in the central region, which is where King Faisal Specialist Hospital and Research Center is located, the financial obstacle was more significant predictor of lower QoL than geographic obstacles (*p* = 0.014). While medical costs are covered by government healthcare, families still incur expenses related to private care, informal support, and special education (14, 46, 47). Moreover, 69.1% of families in our study had a sole provider, coupled with the fact that about half of our cohort had a below-average income (10,000 SAR), this may contribute to suboptimal QoL scores in families with rare diseases.

Caregiving responsibilities disproportionately impact mothers, leading to reduced social participation, job loss, and frequent long-distance hospital visits (48, 49). However, in our study, fathers reported lower QoL than mothers, likely due to financial stress, as most fathers in this study were the sole providers in single-income households half of which having less than average income. The caregiving burden in this context is profoundly intensified by internal family dynamics; notably, 85% of mothers serve as sole caregivers, often managing complex needs for multiple affected individuals, as 50.5% of families reported more than one member with the same diagnosis. Furthermore, the scale of domestic responsibility is underscored by family size, with 86.9% of households supporting more than two siblings with the child having a rare disease. This creates a state of “caregiver saturation” within the primary domestic environment, pushing the caregiver’s physical and emotional capacity to its limits driving the suboptimal QoL scores observed in our cohort.

Support group participation was low in our cohort (10.3%), despite the well-documented benefits of psychosocial support (50, 51). This might be due to cultural differences and different support dynamics. Furthermore, For X-linked conditions like DMD, mothers often face profound “carrier stigma” and internalized guilt regarding the transmission of the mutated gene. Notably, support group participation showed no significant association with QoL in our analysis (*p* = 0.959). However, this result should be interpreted with caution due to the small subgroup size, which may have limited the statistical power to detect a meaningful difference.

Frequent ED visits were strongly associated with lower QoL, with six or more annual visits being a significant predictor in our multi-variate analysis (*p* = 0.003). Many families rely on ED visits due to accessibility barriers to primary physicians or tertiary care centers (52). However, some families prefer to visit their tertiary care center for care due to limited knowledge in rare diseases in primary care hospitals, as suggested by a study in Spain (53). Moreover, 75.7% of our participants lived outside the central region where most tertiary care centers are located adding to the burden of recurrent, lengthy commutes to the ED. Furthermore, geographical barriers such as long distance to a tertiary hospital were cited as a major obstacle by 29.9% of participants and given that A lack of patient education may also contribute to unnecessary ED visits, as improved education has been shown to significantly reduce hospital visits (54, 55). Home healthcare, a well-established strategy for managing chronically ill pediatric patients, has been shown to reduce ED visits while improving family well-being (56, 57).

### Limitations

The study’s cross-sectional design limits the ability to establish causality, and its single-center nature may affect the generalizability of findings to other populations. Moreover, while the three diseases studied differ in clinical trajectories and caregiving challenges, predictors of quality of life were examined across the full sample to maximize statistical power and identify potential shared determinants. This approach may obscure disease-specific effects and should be interpreted with caution. Future studies with larger samples are needed. Furthermore, A limitation of this study is the multiple testing burden in our univariate analysis. With a 19.3% false discovery rate, some individual associations may be false positives. To mitigate this, we prioritized the multivariable stepwise regression results, which provide a more robust identification of independent predictors. In addition to that, the single-informant design might not capture both caregivers’ quality of life, and the use of self-reported measures might introduce recall bias despite only selecting families with a diagnosed child in the last 5 years. Lastly, COVID-19 pandemic might have altered the healthcare and socioeconomic landscape over the past 5 years which might affect caregiver experience. However, the study offers insights into the factors influencing QoL among parents of children with rare diseases, contributing to the literature and highlighting opportunities for targeted interventions and policy improvements.

## Conclusion

Overall, the study found that parents of children with rare diseases experience a suboptimal QoL score, with parents of children with DMD faced the greatest challenges, suggesting targeted interventions like enhanced newborn screening and utilizing genetic testing for expedited diagnosis may be beneficial. The study also found that frequent emergency department visits negatively impacted QoL, suggesting that enhancing healthcare access through medical education, and the integration of telemedicine and home healthcare services might alleviate the burden faced by caregivers. Moreover, financial support to these families might play a role in enhancing their quality of life due to high prevalence of sole providers and caregiver saturation. The low participation in support groups signals a gap in caregiver support that requires further exploration.

## Data Availability

The raw data supporting the conclusions of this article will be made available by the authors, without undue reservation.
